# Accuracy assessment of topography and forest canopy height in complex terrain conditions of Southern China using ICESat-2 and GEDI data

**DOI:** 10.3389/fpls.2025.1547688

**Published:** 2025-03-20

**Authors:** Lianjin Fu, Qingtai Shu, Zhengdao Yang, Cuifen Xia, Xiao Zhang, Yiran Zhang, Zeyu Li, Shengjiao Li

**Affiliations:** ^1^ College of Soil and Water Conservation, Southwest Forestry University, Kunming, China; ^2^ College of Forestry, Southwest Forestry University, Kunming, China; ^3^ Forestry Bureau of Jianhe County, Qiandongnan, Guizhou, China; ^4^ College of Surveying and Mapping and Information Engineering, West Yunnan University of Applied Sciences, Dali, China

**Keywords:** GEDI, ICESat-2, ground elevation, forest canopy height, slope

## Abstract

ICESat-2 and GEDI offer unique capabilities for terrain and canopy height retrievals; however, their performance and measurement precision are significantly affected by terrain conditions. Furthermore, differences in data scales complicate direct comparisons of their measurement capabilities. This study evaluates the accuracy of terrain and canopy height retrievals from ICESat-2 and GEDI LiDAR data in complex terrain environments. Jinghong City and Pu’er City in Southwest China were selected as study areas, with high-precision airborne LiDAR data serving as a reference. Ground elevation and canopy height retrieval accuracies were compared before and after scale unification to 30 m × 30 m under varying slope conditions. Results indicate that ICESat-2 shows a significant advantage in terrain height retrieval, with RMSE values of 4.75 m and 4.21 m before and after scale unification, respectively. In comparison, GEDI achieved RMSE values of 4.94 m and 4.96 m. Both systems maintain high accuracy in flat regions, but accuracy declines with increasing slope. For canopy height retrieval, GEDI outperforms ICESat-2. Before scale unification, GEDI achieved an R² of 0.73 with an RMSE of 5.15 m, and after scale unification, an R² of 0.67 with an RMSE of 5.32 m. In contrast, ICESat-2 showed lower performance, with an R² of 0.65 and RMSE of 7.42 m before unification, and an R² of 0.53 with RMSE of 8.29 m after unification. GEDI maintains higher canopy height accuracy across all slope levels. Post-scale unification, both systems show high accuracy in ground elevation retrieval, with ICESat-2 being superior. In contrast, GEDI achieves better canopy height retrieval accuracy. These findings highlight the synergistic strengths of ICESat-2’s photon-counting and GEDI’s full-waveform LiDAR techniques, demonstrating advancements in satellite laser altimetry for terrain and canopy height retrieval.

## 1 Introduction

In late 2018, NASA initiated two satellite LiDAR missions: the Ice, Cloud, and Land Elevation Satellite-2 (ICESat-2) and the Global Ecosystem Dynamics Investigation (GEDI) (Markus et al., 2017; Dubayah et al., 2020). These missions have produced novel three-dimensional (3D) data of Earth’s surface, providing a unique opportunity to map global variations in topographic features and canopy height (Urbazaev et al., 2022). The main purpose of these data is to deliver accurate vertical measurements, such as topographic elevation (Neuenschwander et al., 2020; Huang et al., 2023a; Li et al., 2023a) and forest canopy height (Potapov et al., 2021; Lang et al., 2022; Mulverhill et al., 2022; Huang et al., 2023b). Topographic elevation is crucial for quantifying glacier elevation changes, mapping forest structure, and estimating biomass (Yang et al., 2011; Mukherjee et al., 2013; Wang et al., 2021). Similarly, the height of the forest canopy plays a crucial role in evaluating forest biomass and carbon sequestration (Zhang et al., 2014; Li et al., 2015; Persson and Perko, 2016). For ICESat-2, the laser footprint has a diameter of approximately 17 meters, with a spacing of about 0.7 meters. Its horizontal accuracy is within 6.5 meters, and the vertical accuracy is 0.1 meters (Magruder et al., 2020, 2021). In contrast, GEDI’s laser footprint has a diameter of approximately 25 meters, with a spacing of around 60 meters. After calibration, GEDI reaches a horizontal positional accuracy of approximately 10.2 meters, with vertical accuracy exceeding 1 meter (Schleich et al., 2023). However, the precision of LiDAR measurements is notably influenced by terrain complexity, particularly in areas with varying slopes (Khosravipour et al., 2015; Goulden et al., 2016; Aryal et al., 2017). Therefore, examining the influence of complex terrain on the measurement precision of GEDI and ICESat-2/ATLAS data is crucial for improving data quality and dependability. Key parameters of ICESat-2 and GEDI are summarized in **Supplementary Table S1** in the **Supplementary Materials**.

Spectral techniques, a cornerstone of remote sensing, are widely applied in soil and terrain studies. For example, Visible-Near Infrared (Vis-NIR) spectroscopy, known for its efficiency, speed, and cost-effectiveness, is a common method for estimating soil organic matter (SOM). Zhang et al. (2021) successfully estimated organic matter in salt-affected soils using Vis-NIR with optimized band combination algorithms, while Zhang et al., (2020a) improved SOM prediction accuracy in Northwest China using fractional-order derivative spectroscopy and modified normalized difference indices. Stevens et al. (2013) and Mohamed et al. (2023) mapped soil organic carbon and soil types using Vis-NIR and hyperspectral remote sensing, respectively. In terrain studies, Dalponte et al. (2015) characterized land cover classification and surface roughness using hyperspectral data. Although spectral techniques excel at analyzing surface material properties (e.g., soil types and vegetation biochemical parameters), they struggle to directly measure three-dimensional structural information (e.g., terrain elevation and canopy height) in complex terrains. For instance, in mountainous areas, terrain shadows, mixed pixels, and vegetation occlusion often reduce spectral data accuracy. In contrast, LiDAR technology, by emitting laser pulses, penetrates vegetation to obtain high-precision terrain and canopy height data, offering clear advantages in complex terrains. However, LiDAR accuracy is also affected by slope, surface roughness, and vegetation density (Zhu et al., 2020). Thus, a detailed evaluation of ICESat-2 and GEDI’s performance in complex terrains not only highlights their advantages over spectral techniques but also offers new insights for integrated terrain and vegetation studies.

The physical mechanisms underlying LiDAR signal degradation in complex terrains are multifaceted. First, topographic slope induces geometric distortion of laser footprints: when the incident angle deviates from nadir, the elliptical footprint expansion causes signal energy dispersion (Hofton et al., 2002; Yeh et al., 2017). For example, a 20° slope can increase ICESat-2’s effective footprint area by 30%, significantly reducing signal-to-noise ratio (Hsu et al., 2021). Second, surface roughness in rugged terrains leads to diffuse reflection effects, where heterogeneous scattering generates mixed waveforms that challenge ground and canopy separation (Neumann et al., 2019). Third, vegetation occlusion in mountainous areas creates “pseudo-ground” errors, as dense canopies obstruct laser penetration to the true terrain surface (Schneider et al., 2019). These mechanisms collectively degrade vertical accuracy.

To mitigate the impact of complex terrain on LiDAR measurement accuracy, extensive research has been conducted to evaluate and improve the performance of ICESat-2 and GEDI in terrain and canopy height retrieval (Urbazaev et al., 2022; Pronk et al., 2024). Zhu et al. (2020) the topographic slope in forested regions was estimated using ICESat-2 data, with results showing higher accuracy compared to SRTM DEM and GDEM data. Similarly, Geng et al. (2021) employed ICESat-2 data to produce a Digital Elevation Model (DEM) of the Antarctic ice shelves, achieving higher spatial resolution and elevation accuracy. Neuenschwander and Magruder (2019) evaluated ATL08 canopy height data products against airborne LiDAR measurements from vegetated areas in Finland, reporting an R² of 0.98 and an RMSE of 3.69 m. Pang et al. (2022) It was shown that integrating high-accuracy Digital Terrain Models (DTM) with ATLAS products substantially enhances the precision of canopy height retrieval in mountainous forest environments, with correlation coefficients (R) between ATLAS-derived canopy heights and corresponding ALCH values ranging from 0.61 to 0.94, depending on segment lengths of 20, 60, and 100 meters. Zhu et al. (2023) employed airborne LiDAR data from the Harvard Forest to assess the precision of GEDI measurements for both ground elevation and canopy height, yielding RMSE of 9.76 meters for elevation and 5.50 meters for canopy height. Juan et al (Guerra-Hernández and Pascual, 2021). evaluated the accuracy of GEDI ground elevation data by comparing it to airborne LiDAR measurements collected in Spain, achieving an RMSE of 4.48 meters. Qi and Dubayah (2016) integrated simulated GEDI data with TANDEM-X InSAR to produce maps of forest structural characteristics. Fayad et al. (2021b) evaluated canopy height in Brazilian eucalyptus plantations using GEDI data, finding that stepwise regression analysis provided relatively precise estimates in flatter areas, with an RMSE of 1.33 meters.

While prior research has explored the effectiveness of GEDI and ICESat-2 for terrain and canopy height retrieval, limitations persist in fully understanding their performance within complex terrain. For example, some studies have focused on relatively simple terrain, which may not fully represent the challenges inherent in complex topography. Furthermore, there remains a need for robust validation datasets and more comparative studies of ICESat-2 and GEDI performance across diverse terrain conditions. To address these limitations, the present study focuses on the complex terrain of Jinghong and Pu’er cities in Southwest China. Utilizing high-resolution airborne LiDAR data as a reference, the effectiveness of satellite LiDAR data—GEDI and ICESat-2—in retrieving terrain and canopy height at a consistent spatial scale is evaluated. This research aims to enhance the reliability of remote sensing data and provide a scientific basis for the accurate estimation of forest biomass and structure. Moreover, this study endeavors to bridge the gap in comprehensively assessing the performance of GEDI and ICESat-2 data under complex terrain conditions, thereby offering a crucial reference benchmark for future research and applications in related fields.

## 2 Materials and methods

### 2.1 Study area

To assess the accuracy of two satellite LiDAR datasets in extracting ground elevation and forest canopy height, this study selected three study sites located in two regions of Yunnan Province, China—Jinghong and Pu’er cities—characterized by their complex terrain and diverse forest ecosystems (Wang et al., 2022b; Guan et al., 2024). Based on the quantitative analysis methods for terrain complexity from Xiong et al. (2022) complex terrain in this study is quantitatively defined by the following three key indicators: elevation range, slope gradient distribution (percentage of area with slopes > 25°), and terrain ruggedness index (TRI). Jinghong City, situated in the southwest of Yunnan Province (100°25′E-101°31′E, 21°27′N-22°36′N), exhibits moderate terrain complexity with elevations ranging from 485 to 2196.8 m, an average slope of 18.8 degrees, and 23.1% of the area having slopes > 25° (mean TRI value: 1.29). This experimental area, characterized by steep valley systems and undulating hill sequences, was specifically selected for DEM extraction comparison due to its distinct topographic features that present challenges for satellite LiDAR ground detection algorithms. Located in southern Yunnan Province (99°09′E-102°19′E, 22°02′N-24°50′N), Pu’er City exhibits higher terrain complexity with elevations spanning from 317 to 3370 m, an average slope of 21.6 degrees, and approximately 35.0% of the region having slopes > 25° (mean TRI value: 1.45). This area was chosen for Canopy Height Model (CHM) extraction analysis due to its complex topography and diverse forest cover (62.8% of the total area).

#### 2.2.1 ICESat-2 spaceborne lidar data

This study utilized Earthdata Search (https://earthdata.nasa.gov/) to acquire and process both ICESat-2 ATL03 geolocated photon data and ATL08 land and vegetation height datasets. Unlike band selection and dimensionality reduction strategies commonly employed in spectral data processing, LiDAR data separates terrain and vegetation signals by analyzing photon density and distribution characteristics (Zhang et al., 2020b). The ATL08 product is generated through processing of the ATL03 raw photon data, with its workflow first employing the Differential, Regressive, and Gaussian Adaptive Nearest Neighbor (DRAGANN) algorithm to remove noise from the ATL03 photon point cloud. This algorithm iteratively analyzes photon density and range characteristics within spatial neighborhoods, and more effectively distinguishes signal photons representing vegetation and ground reflections from background noise compared to traditional fixed-window photon filtering methods (Neuenschwander and Magruder, 2019; Kui et al., 2023). Following noise removal, the official land and ice surface classification method developed by the NASA ICESat-2 science team is applied to the photon classification process. This method precisely differentiates various surface features and estimates vegetation canopy height through predefined thresholds and rules based on photon characteristics and auxiliary data, categorizing signal photons into canopy top photons, canopy photons, ground photons, and noise photons (Zhu et al., 2022; Feng et al., 2023). Through these processing steps, the resulting ATL08 product provides multiple parameters related to vegetation canopy and terrain for each 100-meter segment along the satellite track, including ground elevation, canopy height, measurement uncertainty, slope, signal-to-noise ratio, and cloud cover rate (Neuenschwander and Pitts, 2019; Osama et al., 2024). Parameters extracted from ATL08 and ATL03 data products are summarized in **Supplementary Table S2** of the **Supplementary Materials**.

A geospatial matching algorithm (Zhu et al., 2022) implemented in Python was utilized to link the ATL03 and ATL08 datasets. By using group IDs (ph_segment_id = segment_id), the algorithm identifies the starting photon index of each group (ph_index_beg). Combining this information with the relative photon index within the group (classed_pc_indx), the algorithm calculates the global photon index in ATL03 as classed_pc_indx + ph_index_beg - 1. This enables precise mapping of photons between ATL08 and ATL03, thereby extracting the spatial distribution and classification information of photons within each statistical unit. To ensure data availability, timeliness, and temporal consistency between airborne and spaceborne LiDAR data, the acquisition timing of the airborne LiDAR data was carefully considered in this research. This is crucial to avoid inaccurate forest canopy height extraction caused by temporal differences between datasets. Following spatial filtering and data quality checks of 64 HDF5 files, 762 valid measurement samples were successfully obtained. Data acquisition periods were from September 2021 to September 2023 for the Pu’er study area, and from February to December 2019 for the Jinghong study area.

#### 2.2.2 GEDI L2A spaceborne lidar data

**Figure 1:**
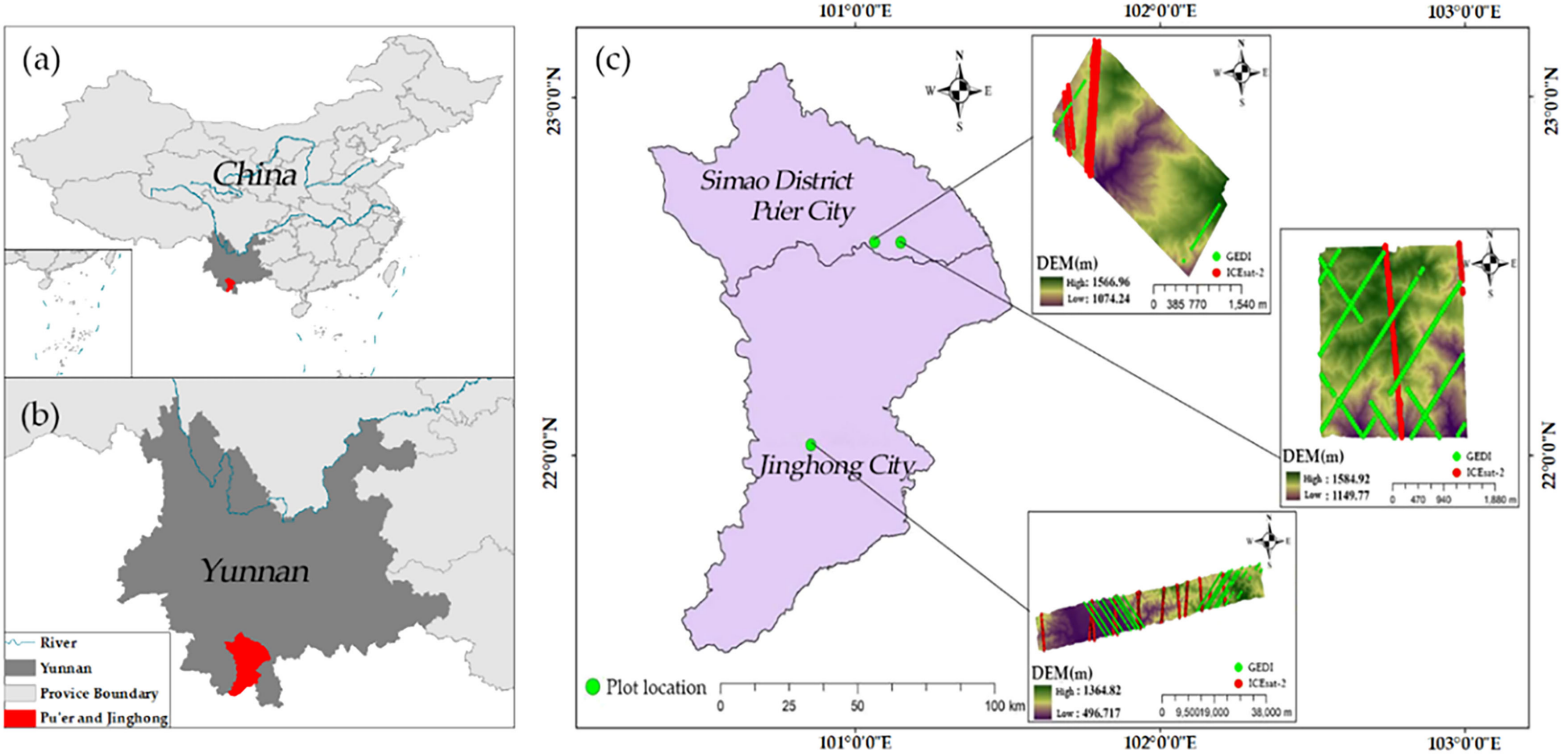
Study area. **(a)** Location of Pu’er and Jinghong in China. **(b)** Location of Pu’er and Jinghong in Yunnan Province. **(c)** Distribution of spaceborne LiDAR tracks, airborne LiDAR data coverage, and sampling points within the study area. The background displays a DEM generated from airborne LiDAR data. Red lines: ICESat-2 LiDAR tracks; Green lines: GEDI LiDAR tracks; Green dots: Airborne LiDAR sampling point locations. Airborne LiDAR data coverage is indicated by the DEM background map.

The GEDI L2A data product is capable of extracting canopy height and ground elevation parameters at the footprint scale (Wang et al., 2022a; Li et al., 2024a), which aligns with the conditions of the complex terrain study and the objectives to assess the accuracy of LiDAR datasets in extracting topography and forest canopy height parameters. GEDI L2A data products corresponding to the study area were selected for analysis based on the study region and the time range of the airborne LiDAR data, with data from the same period as ICESat-2. The GEDI L2A data covering the study area were downloaded via Earthdata Search (Earthdata Search | Earthdata Search (nasa.gov)), resulting in a total of 837 footprint data points. **Figure 1** illustrates the spatial distribution of the data points. Each GEDI L2A data product includes 8 beams, with each beam containing approximately 156 parameter fields. This study primarily extracted 11 key fields, including footprint quality, geolocation, ground elevation, and canopy height parameters, which are described in **Supplementary Table S3** in the **Supplementary Materials**. To ensure data quality, this study implemented a quality filtering process based on the steps outlined in reference (Li et al., 2024b) to obtain high-quality GEDI footprint data for analysis. Specifically, the downloaded GEDI L2A data were initially spatially clipped to the extent of the airborne LiDAR data within the study area to extract footprints within the region of interest. Subsequently, quality parameters provided within the GEDI L2A product were utilized for filtering. Only footprints meeting the following quality criteria were retained: = 1 (valid waveform), = 0 (pointing and geolocation information not degraded), sensitivity ≥ 0.9 (good footprint quality), and rx_assess_flag = 0 (no significant waveform analysis errors). Footprint data not conforming to these quality standards were excluded.

#### 2.2.3 Airborne LiDAR data

**Figure 2:**
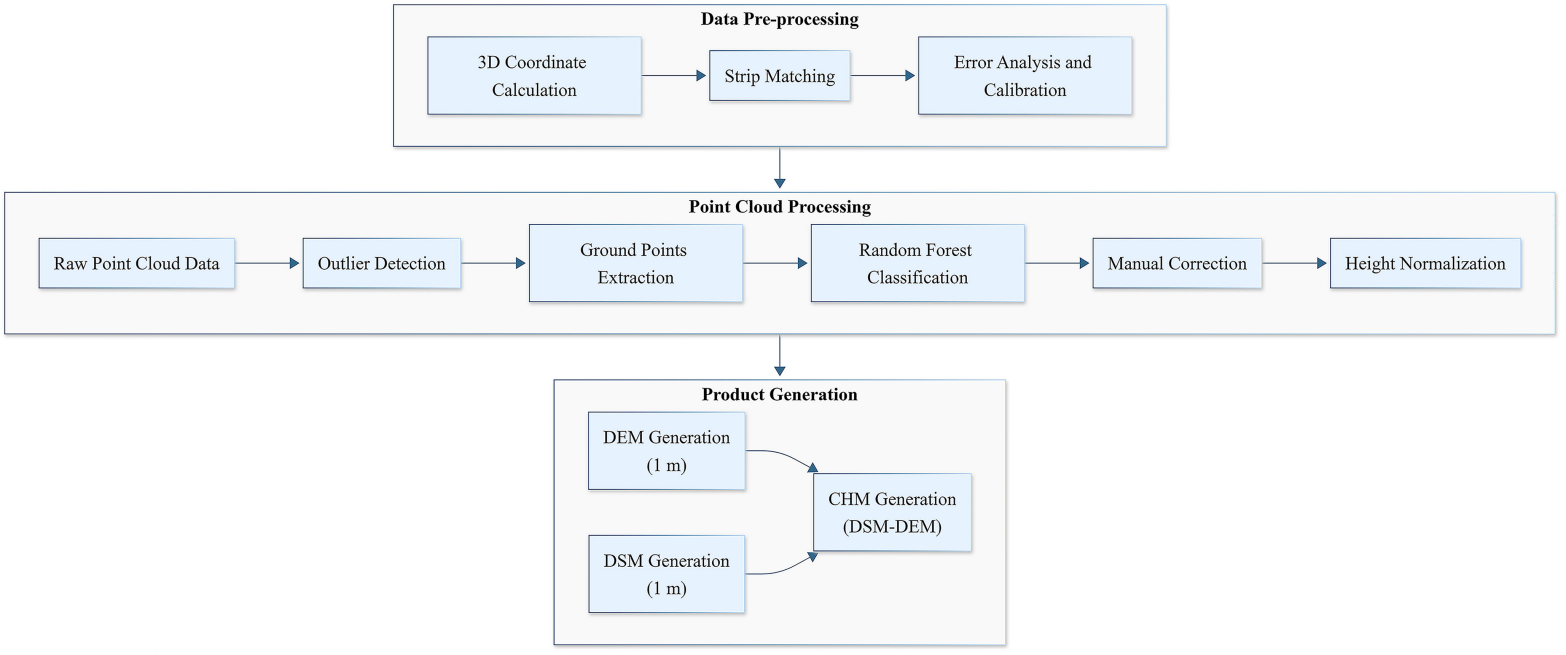
Airborne lidar point cloud data processing workflow.

The capability of airborne LiDAR data in detecting forest spatial structure and understory topography has been widely acknowledged (Lu et al., 2016; Ma et al., 2017). Consequently, this study utilizes airborne LiDAR data as reference data to evaluate spaceborne LiDAR data products. The airborne LiDAR data for Jinghong and Pu’er cities in Yunnan Province were obtained from the LiCHy (LiDAR, CCD, and Hyperspectral) system of the Chinese Academy of Forestry (**Figure 1**). For Jinghong City, the LiDAR data were acquired in April 2014, covering an area of approximately 119.78 km². In contrast, the LiDAR data for Pu’er City were collected from December 2022 to January 2023, covering areas of 13.08 km² and 6.52 km², respectively. Both datasets utilized the RIEGL LMS-Q680i sensor. The average flight altitude was about 851 meters, with a scan angle set to ±30 degrees and a maximum pulse frequency of 400 kHz. The point cloud density was 3.9 points per square meter, and the data were saved in LAS 1.2 format. This study employed a comprehensive airborne LiDAR data processing workflow (**Figure 2**) encompassing initial coordinate calculation, flight strip alignment, and system calibration, with subsequent steps including point cloud denoising, classification of ground and vegetation points, manual refinement editing, and elevation normalization. Traditional workflows, often relying on single filtering algorithms, are prone to misclassification in areas with significant topographic relief (Pingel et al., 2013), which notably reduces the accuracy of DEMs and CHMs. In contrast, this workflow integrated a statistical outlier detection algorithm (Hodge and Austin, 2004) and a random forest classification algorithm (Denisko and Hoffman, 2018). To further enhance accuracy, especially in topographically complex areas, a manual refinement editing step was introduced to correct classification results and mitigate error accumulation. This refined workflow ultimately generated 1-meter resolution DEM and CHM products.

### 2.3 GEDI geographic location error correction

Studies (Liu et al., 2021; Tang et al., 2023; Pronk et al., 2024) indicate that ICESat-2 achieves horizontal geolocation accuracy within 5 meters, with positional errors typically ranging between 2 and 3 meters. Comparatively, the first version of GEDI data has a positional error of approximately 20 meters, whereas the second version reduces this error to 10.2 meters. Compared to ICESat-2, GEDI has more significant horizontal positioning errors, which could impact the accuracy of studies that directly use the centroid coordinates of GEDI footprints. For applications requiring extremely high elevation accuracy, GEDI’s terrain height measurements are likely significantly influenced by its geolocation errors, making it a notable source of error. Similarly, in studies of canopy height inversion or ground elevation extraction, horizontal positioning errors significantly affect accuracy evaluations. Given that GEDI data products have room for further optimization, geolocation error correction must be performed before validating ground elevation and forest canopy height accuracy.

**Figure 3:**
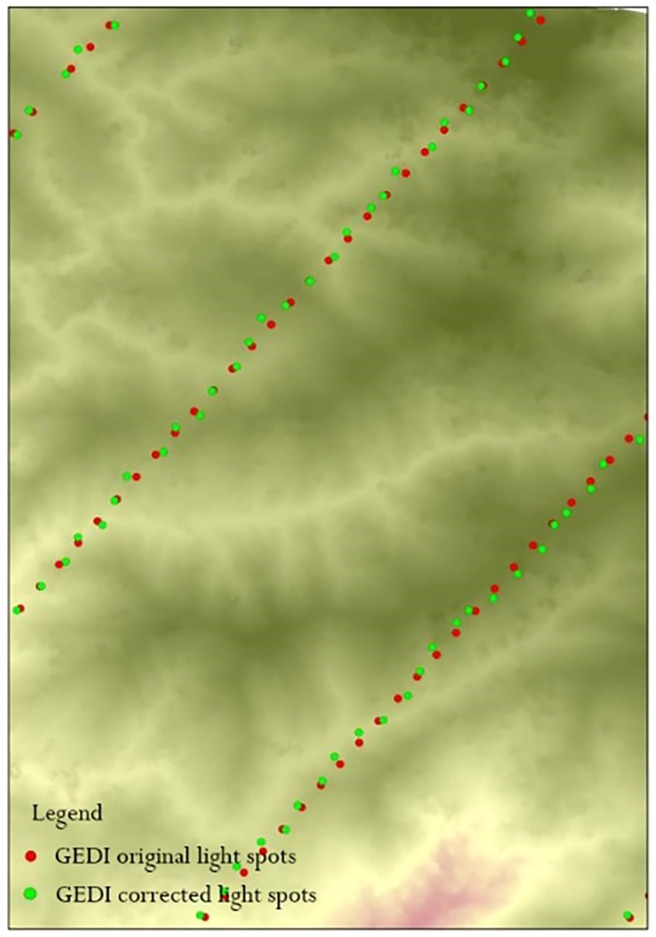
Map of spot location before and after GEDI location error correction (part).


Based on waveform simulation techniques proposed by (Blair and Hofton, 1999), several studies (Neuenschwander et al., 2008; Milenković et al., 2017; Hancock et al., 2019) have explored converting discrete return data from airborne LiDAR systems (ALS) into simulated wide-area, full-waveform LiDAR information. These techniques vary in two key aspects: the simulation of footprint intensity patterns and the weighting approach applied to ALS point contributions. In this study, the approach outlined by (Hancock et al., 2019) was utilized. Using the GEDI simulator, researchers modified specific parameters to align with different airborne LiDAR data sets and generate simulated GEDI waveform data. For each GEDI footprint, a search was first conducted within a 25-meter diameter range around the centroid coordinates with a 1-meter step size. Airborne LiDAR data were subsequently utilized to generate simulated GEDI waveforms, and the Pearson correlation coefficient was computed to compare these simulated waveforms with the GEDI L1B waveforms. The waveform with the highest correlation coefficient was identified, and its corresponding position was taken as the corrected true position of the GEDI footprint (**Figure 3**). The calculation formula is as follows:

$$I_{w,i}{=I}_{i}\frac{1}{\sigma_{f}\sqrt{2\pi }}e^{\frac{{\left(x_{i}{-x}_{c}\right)}^{2}+{\left(y_{i}{-y}_{c}\right)}^{2}}{{2\sigma }_{f}^{2}}}$$

$$WV\left(z\right)=\sum_{i=1}^{N}I_{w,i}*\frac{1}{\sigma_{p}\sqrt{2\pi }}e^{\frac{{\left({z-z}_{i}\right)}^{2}}{{2\sigma }_{p}^{2}}}$$

In the equation, $I_{w,i}$ represents the energy intensity value of the i-th pulse echo, $I_{i}$ is the original intensity value of the i-th point cloud, $\sigma_{f}$ denotes the standard deviation of the Gaussian distribution of footprint energy, ( $x_{i}$ , $\;y_{i}$ ) denotes the position of the I-th point cloud, ( $x_{c}$ , $\;y_{c}$ ) represents the central position of the simulated waveform corresponding to the footprint, WV(z)  is the intensity value of the simulated waveform at height z, N is the number of point clouds, ∗ denotes the convolution operation, and $\sigma_{p}$ is the standard deviation of the emitted pulse width.

### 2.4 Experimental design of spaceborne lidar data product accuracy verification

This research focused on analyzing how varying slope conditions affect the accuracy of two satellite LiDAR data products. The terrain slopes within the study area were classified into six categories: Flat slope (<5°), Gentle slope (5-15°), Moderate slope (15-25°), Steep slope (25-35°), Extremely steep (35-45°), and Dangerous slope (>45°). However, the resolution differences between the satellite LiDAR data products (ICESat-2 and GEDI) and the airborne LiDAR data make direct comparisons of elevation information challenging. Therefore, this study employed a four-step validation methodology: (1) generating buffer zones based on data product fragment sizes and footprint coverage areas; (2) extracting ground elevation and canopy height information within the corresponding buffer zones; (3) converting vertical reference planes for terrain height; and (4) evaluating accuracy.

ICESat-2 ATL08 and GEDI L2A data products are stored in HDF5 file format. The ATL08 data product samples and records information in fixed 100m × 12m sections along the track, while the GEDI L2A data product samples using 25-meter circular footprints at 60-meter intervals. For the ICESat-2 ATL08 data product, the center positions of ATL08 data sections were first extracted and unified into the UTM projection coordinate system used by the airborne LiDAR data. Next, the inclination angle of the track was determined by calculating the centroid positions of the start and end points of the track within the test area. A 100 m × 12 m rectangular buffer zone was then created around these positions. For the GEDI L2A data product, a 25-meter diameter circular buffer zone was generated based on the footprint center coordinates. Airborne LiDAR data DEM and CHM products for the corresponding buffer zones were read to calculate the average ground elevation and slope within those zones. Research has suggested (Adrah et al., 2021; Li et al., 2023b; Ngo et al., 2023) that due to the signal noise uncertainty at the canopy top, RH98 should be used as the measurement for canopy top height instead of the maximum value of canopy photons. Therefore, in this study, for each buffer zone generated by the satellite LiDAR centroids, the crown height values of each airborne LiDAR grid within the buffer zone were extracted and ranked, and the 98th percentile (RH98) was calculated to verify the accuracy of forest canopy height. When performing consistency comparisons of elevation data, all heights need to refer to a unified vertical reference plane. In this study, both the satellite LiDAR and airborne LiDAR data use the WGS84 ellipsoid as the vertical reference plane, so no conversion is required. To calculate the RH98 and mean ground elevation, the following formulas were used:


$${CH}_{i}{=HC}_{i}{-HD}_{i}$$

Where ${CH}_{i}$ represents the canopy height for the i-th vegetation point, ${HC}_{i}$ represents the height of the i-th vegetation point from the Digital Surface Model (DSM) representing the canopy top and other surface features, and ${\mathrm{HD}}_{i}$ represents the height of the surface corresponding to the i-th vegetation point from the DEM, representing the bare ground.


$$H_{mean}=\sum_{i=1}^{\text{n}}{(HD}_{i})/n$$

Where $H_{mean}$ represents the average ground elevation within the buffer zone, ${HD}_{i}$ represents the height of the i-th ground point within the buffer zone from the DEM, and n represents the total number of ground points within the buffer zone.


DH=sort(CH)

Where DH represents a dataset formed according to the ascending order of all vegetation points relative to the ground height, CH represents the dataset containing all canopy height values of vegetation points, and sort (CH) denotes the operation of sorting the dataset CH in ascending order.


$${RH}_{\text{j}}=DH(i),\;j=98$$

i=0.01jn

Where ${RH}_{\text{j}}$ represents the j-th percentile of the canopy height, DH represents the canopy height dataset sorted in ascending order, j represents the percentile value, n  represents the total number of vegetation points within the buffer zone, i represents the index position of the RH_j_ value in the DH dataset, and DH(i) represents the i-th element’s value extracted from the sorted dataset DH .

### 2.5 Design of unified scale accuracy verification experiment

This study utilizes ICESat-2’s ATL08 and ATL03 data to generate DEM and CHM. A uniform scale of 30 m × 30 m was selected for processing both ICESat-2 and GEDI data, based on several key factors: (1) It closely aligns with GEDI’s laser footprint diameter of 25 m while providing a slight buffer to account for geolocation uncertainty; (2) For the regional scale of this study, a 30 m resolution has been deemed sufficient to capture the main terrain and forest canopy characteristics in the Jinghong and Pu’er regions, as finer resolutions are unlikely to yield significant accuracy improvements while substantially increasing computational demands and data volume; and (3) In the context of accuracy comparison between ICESat-2 and GEDI data, the uniform adoption of a 30 m resolution ensures spatial consistency and facilitates direct performance evaluation at a comparable scale.

For DEM generation, ground photons (classified as “1”) were extracted from ATL08 and ATL03 data. Using ArcGIS, a 30 m × 30 m grid was created, and the average elevation of ground photons within each grid cell was calculated to determine the ICESat-2 DEM value. To ensure robust elevation estimates, a quality control threshold requiring a minimum of four ground photons per grid cell was implemented; cells not meeting this criterion were excluded from the analysis. For CHM generation, the same ATL08 and ATL03 datasets were utilized to extract multiple photon classifications, including ground, canopy, and top-of-canopy photons. Canopy height measurements were derived at 30 m intervals, with canopy photon heights calculated relative to the mean ground photon height within each grid cell. This process yielded various percentile canopy height parameters (rh80, rh85, rh90, rh95, rh98, and rh100). Quality control measures required the simultaneous presence of canopy top, canopy, and ground photons within each grid cell for valid height calculations.

### 2.6 Accuracy verification

This study primarily uses the DEM and CHM derived from airborne LiDAR data in the study area as the ground truth for accuracy validation. The aim is to assess the accuracy of DEM and CHM extracted from two satellite-based LiDAR sources, ICESat-2 and GEDI, and to explore how slope factors influence this accuracy. The accuracy is evaluated using R² (coefficient of determination) and RMSE (root mean square error), with higher R² values and lower RMSE values indicating better accuracy. The calculation formulas are as follows:


$$R^{2}=1-\frac{\sum_{i=1}^{n}{\left(y_{i}-{\hat{y}}_{i}\right)}^{2}}{\sum_{i=1}^{n}{\left(y_{i}-\bar{y}\right)}^{2}}$$

$$RMSE=\sqrt{\frac{1}{n}\sum_{i=1}^{n}{\left(y_{i}-{\hat{y}}_{i}\right)}^{2}}$$

In the formula, ${\hat{y}}_{i}$ is the ground elevation DEM or forest canopy height CHM of the spaceborne LIDAR; $y_{i}\;$ is the DEM or CHM reference value extracted by the corresponding airborne LIDAR; $\bar{y}\;$ is the mean value of DEM or CHM reference value; n is the number of spaceborne lidar samples.

### 2.7 Mapping of forest canopy height with 30m resolution in the study area

First, 181 GEDI L2A footprint measurements were collected and analyzed using GS+ 9.0 software for variogram analysis. The analysis revealed spatial variations in canopy height across different locations, which led to the selection of the most suitable variogram model based on the observed variability. Ordinary kriging was then used to estimate canopy heights at unmeasured locations based on known measurements and the variogram. The canopy height (RH 90) derived from GEDI L2A footprints, used in the variogram analysis, was incorporated as conditional data in the kriging simulation. After several iterations, the optimal number of simulations was determined to enhance the accuracy of the canopy height estimates. Ultimately, sequential Gaussian simulation was applied to generate the canopy height map for the study area.

## 3 Results and analysis

### 3.1 Ground height retrieval and slope influence

**Figure 4:**
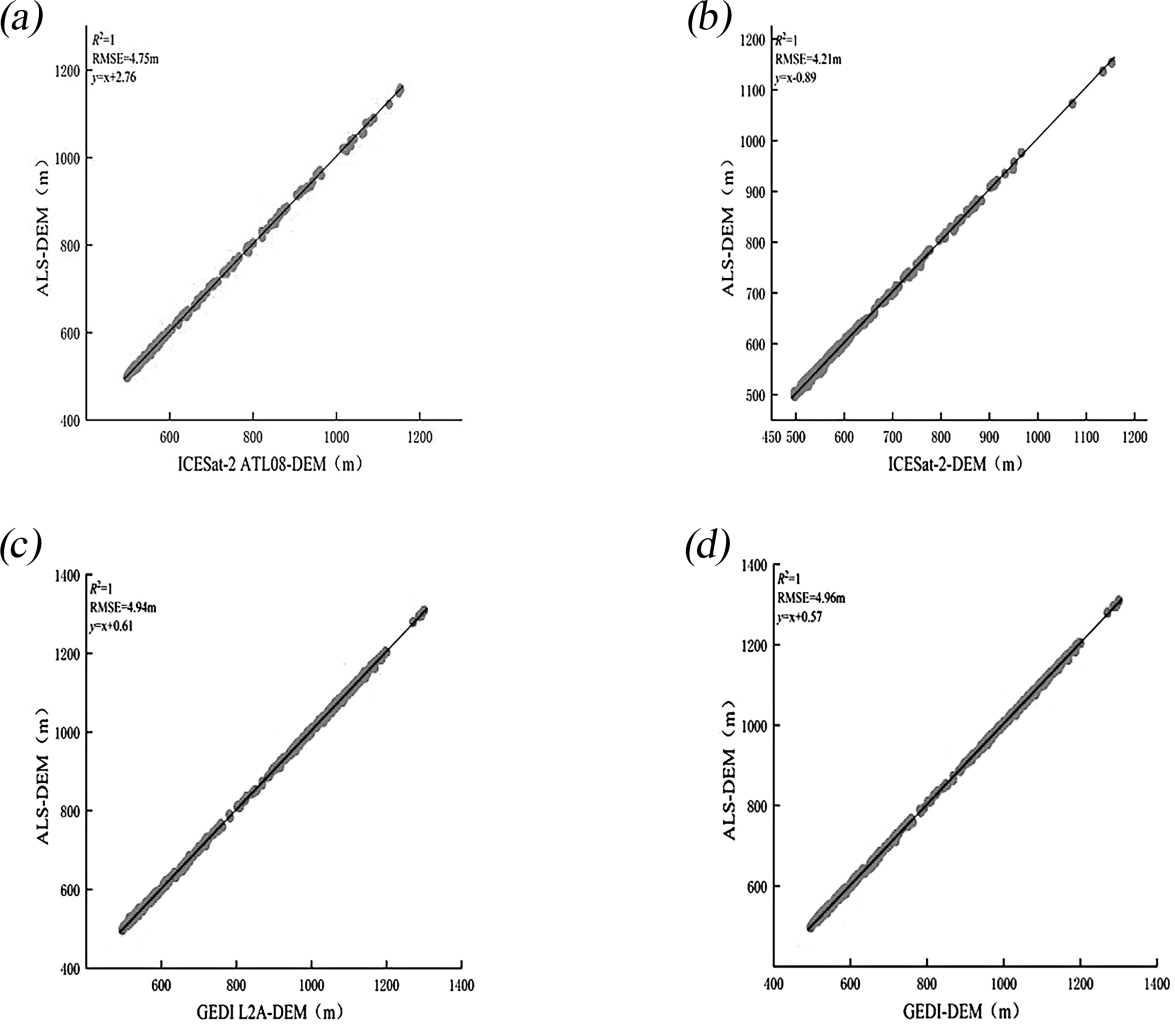
Scatter plots of DEMs show comparisons between ICESat-2 and airborne LiDAR **(a)** and **(b)**, and between GEDI and airborne LiDAR **(c)** and **(d)**, both before and after scale unification.

Airborne LiDAR data provided by the Chinese Academy of Forestry facilitated the validation of terrain and canopy height measurements obtained from ICESat-2 and GEDI across diverse slopes. In the region depicted in **Figure 1**, terrain and canopy heights extracted from the ATL08 (ICESat-2) and L2A (GEDI) products were compared against measurements derived from airborne LiDAR data. For terrain height validation, samples from ICESat-2 and GEDI were analyzed. The exceptionally high R² values (**Figure 4**) demonstrate strong consistency between terrain heights from ICESat-2 and GEDI and those from airborne LiDAR, both before and after scale unification. Additionally, the RMSE of ICESat-2 decreased from 4.75 to 4.21 meters, while GEDI’s RMSE slightly increased from 4.94 to 4.96 meters.

**Figure 5:**
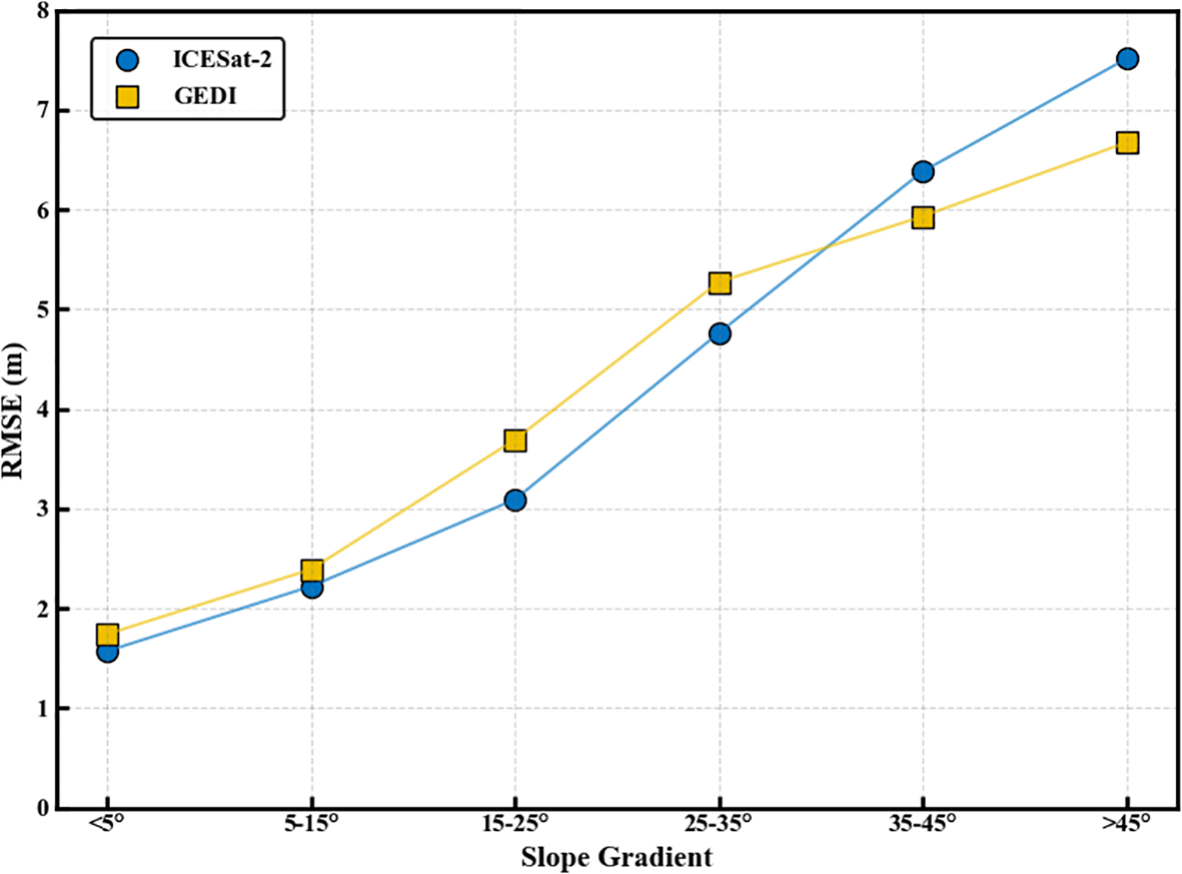
DEM RMSE variation with slope graph.

In previous studies, data from high-relief areas were often less focused on. However, this research incorporates all samples, including those from steep and uneven terrains, aiming to deliver a thorough assessment of the terrain height products from ICESat-2 and GEDI across diverse topographic conditions. Although this approach may introduce higher errors, it ensures a more representative assessment compared to filtered datasets. This study specifically examines the impact of slope on DEM accuracy using LiDAR data. **Figure 5** and **Table 1** display the terrain elevation data for ICESat-2 and GEDI across six distinct slope conditions following scale normalization, highlighting the impact of slope on the precision of terrain height measurements. The results indicate that slope significantly impacts the accuracy of DEMs for ICESat-2 ATL08 and GEDI L2A data. As slope increases, both datasets show a gradual increase in RMSE, indicating a decline in accuracy. Notably, GEDI demonstrates better ground elevation accuracy than ICESat-2 when the slope is greater than or equal to 35°. However, ICESat-2 overall performs better in terrain height retrieval, with an RMSE of 4.21 m compared to GEDI’s RMSE of 4.96 m.

**Table 1:** Results of DEM accuracy verification for different slopes after scale unification.

Gradient Scale	ICESat-2		GEDI	
	R^2^	RMSE/m	R^2^	RMSE/m
Flat slope (<5°)	1.00	1.57	1.00	1.74
Gentle slope (5-15°)	1.00	2.22	1.00	2.39
Moderate slope (15-25°)	1.00	3.09	1.00	3.69
Steep slope (25-35°)	1.00	4.76	1.00	5.27
Extremely steep (35-45°)	1.00	6.38	1.00	5.93
Dangerous slope (>45°)	1.00	7.51	1.00	6.67

### 3.2 Canopy height verification and slope effect

Since the techniques for measuring forest canopy height utilized by GEDI and ICESat-2 ATLAS vary from those applied in drone-based LiDAR, this research contrasts the RH metrics derived from GEDI and ICESat-2 ATLAS with forest canopy height data collected through airborne LiDAR. The objective is to identify the most effective RH metric for representing forest canopy height as derived from GEDI or ICESat-2 ATLAS. When compared to the forest canopy heights from drone LiDAR (**Table 2**), both GEDI’s RH90 and ICESat-2 ATLAS’s RH90 exhibit the highest R² values and the lowest RMSEs. Consequently, these metrics were selected to represent the forest canopy heights obtained from GEDI and ICESat-2 ATLAS.

**Table 2:** Comparison of RH metrics from GEDI and ICESat-2 with airborne LiDAR CHM.

Spaceborne platform	Evaluation indicator	RH80	RH85	RH90	RH95	RH98	RH100
GEDI	R^2^	0.65	0.67	0.73	0.64	0.61	0.61
	RMSE(m)	6.80	5.98	5.15	5.56	5.59	6.19
ICESat-2	R^2^	0.42	0.46	0.53	0.50	0.47	0.45
	RMSE(m)	10.47	9.57	8.29	8.67	9.26	9.73

**Figure 6:**
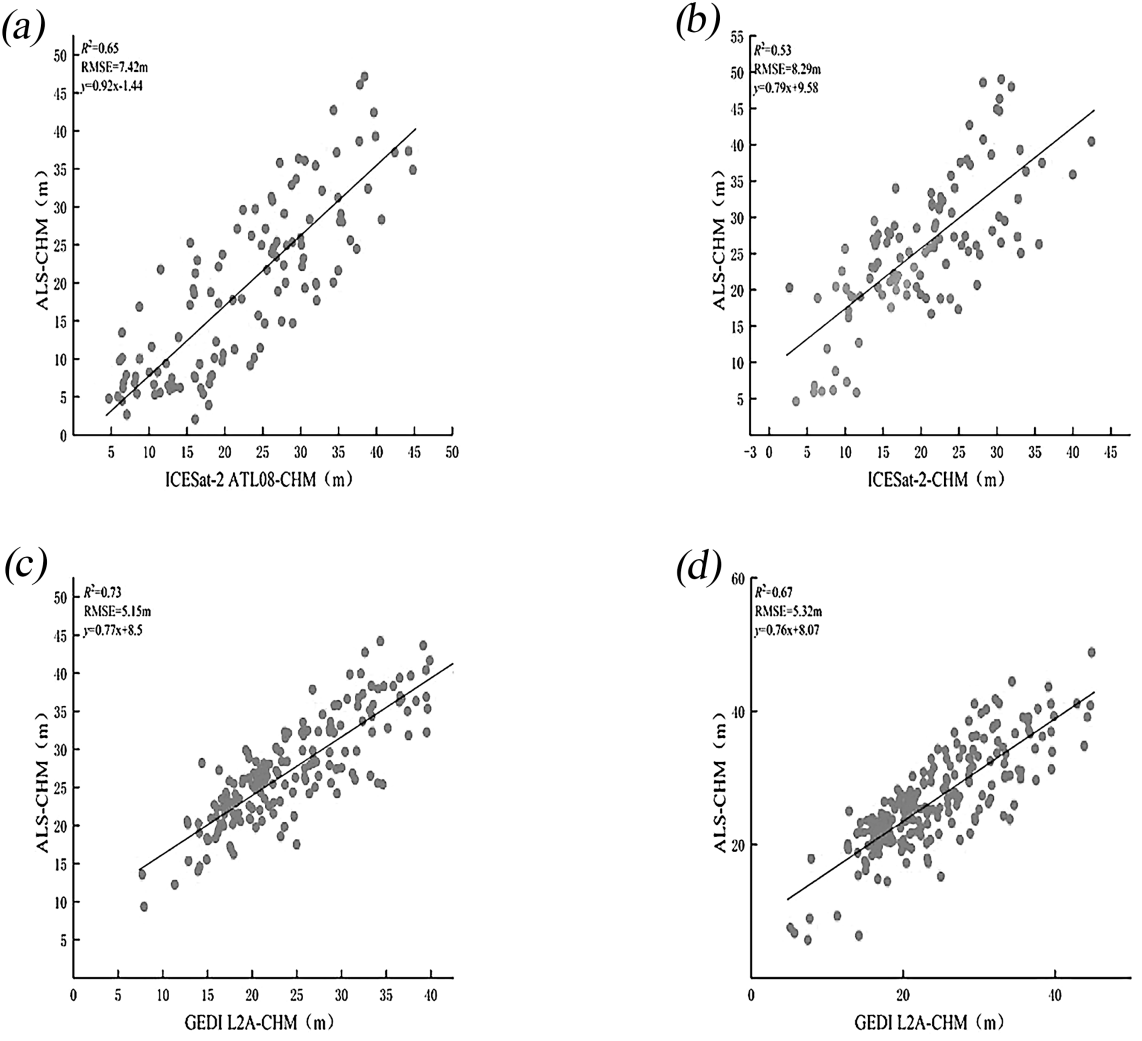
Scatter plots of CHM show comparisons between ICESat-2 and airborne LiDAR **(a)** and **(b)**, and between GEDI and airborne LiDAR **(c)** and **(d)**, both before and after scale unification.

**Figure 6** presents scatter plots illustrating the inversion accuracy of CHM for GEDI and ICESat-2, both before and after scale unification. Following scale unification, the results for ICESat-2’s canopy inversion accuracy indicate a decrease in R² from 0.65 to 0.53 and an increase in RMSE from 7.42 m to 8.29 m. For GEDI, the R² value decreased from 0.73 to 0.67, and the RMSE increased from 5.15 m to 5.32 m. The increase in forest canopy height error may be attributed to inaccuracies in terrain height, which, in turn, affect canopy height accuracy. The results indicate that GEDI’s forest canopy height inversion accuracy is superior to that of ICESat-2, demonstrating better performance in forest parameter inversion. Additionally, similar to ICESat-2 ATL08, the GEDI L2A data demonstrate that the inversion accuracy for DEM is higher than that for CHM.

#### 3.2.1 Effect of slope on CHM accuracy before scale unification

**Figure 7:**
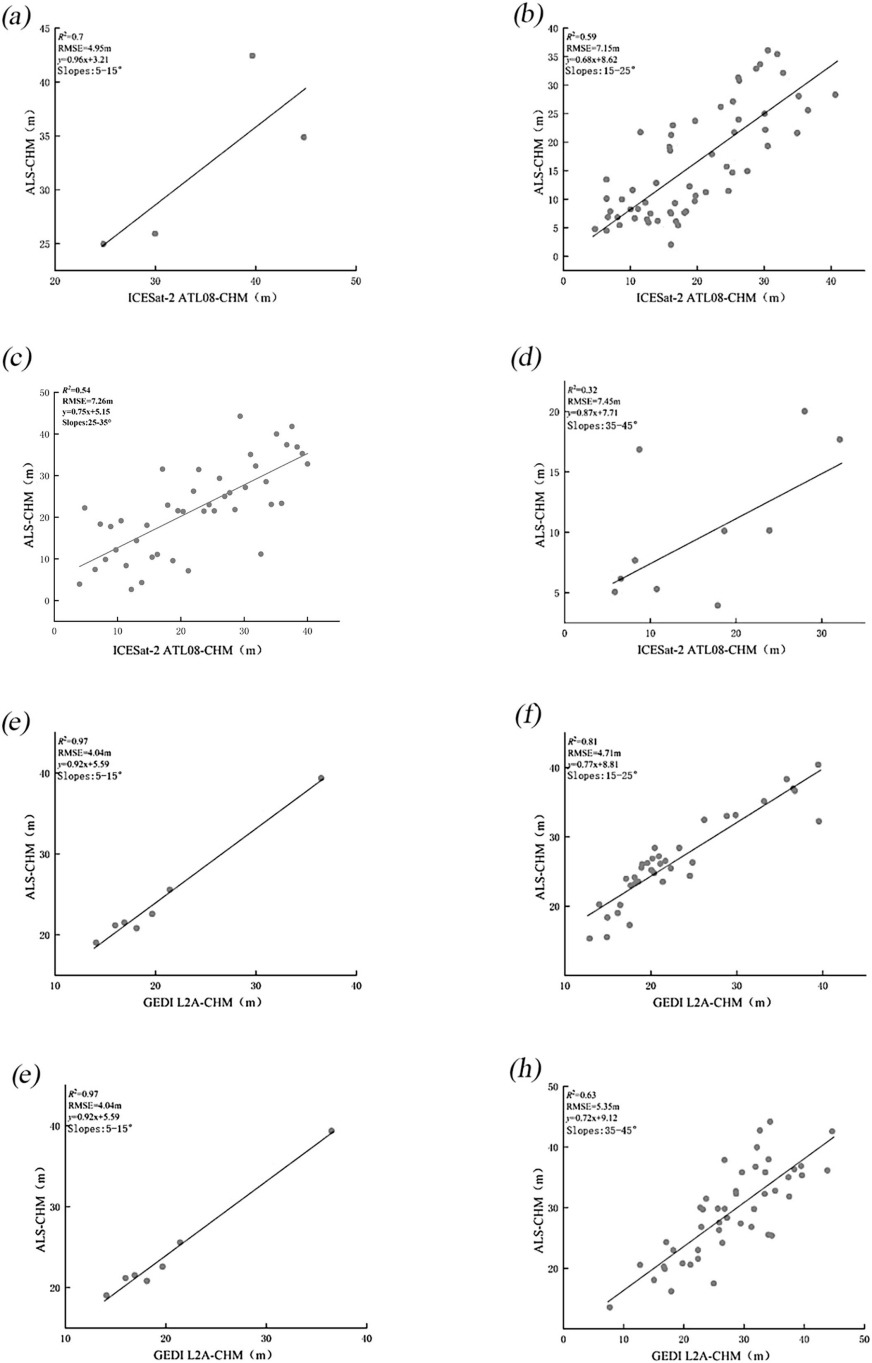
Scatter plots of CHM from ICESat-2 **(a–d)** and GEDI **(e–h)** compared to airborne LiDAR before scale unification, across gentle, moderate, steep, and extremely steep.

This section classifies the study area according to slope in order to explore how different slope conditions affect the accuracy of the CHM generated from two spaceborne LiDAR datasets. The findings (**Figure 7**, **Table 3**) reveal that slope plays a crucial role in determining the CHM precision for both ICESat-2 and GEDI. As slope increases, R² values decrease, and RMSE values increase, reflecting a trend of decreasing accuracy. Furthermore, compared to GEDI L2A, the CHM accuracy of ICESat-2 ATL08 is more strongly influenced by slope.

**Table 3:** Results of CHM accuracy verification at different slopes.

Gradient Scale	ICESat-2		GEDI	
	R2	RMSE/m	R2	RMSE/m
Gentle slope (5-15°)	0.69	4.95	0.97	4.04
Moderate slope (15-25°)	0.59	7.15	0.81	4.71
Steep slope (25-35°)	0.58	7.42	0.72	5.17
Extremely steep (35-45°)	0.32	7.45	0.63	5.35

#### 3.2.2 Effect of slope on CHM accuracy after scale unification

**Figure 8:**
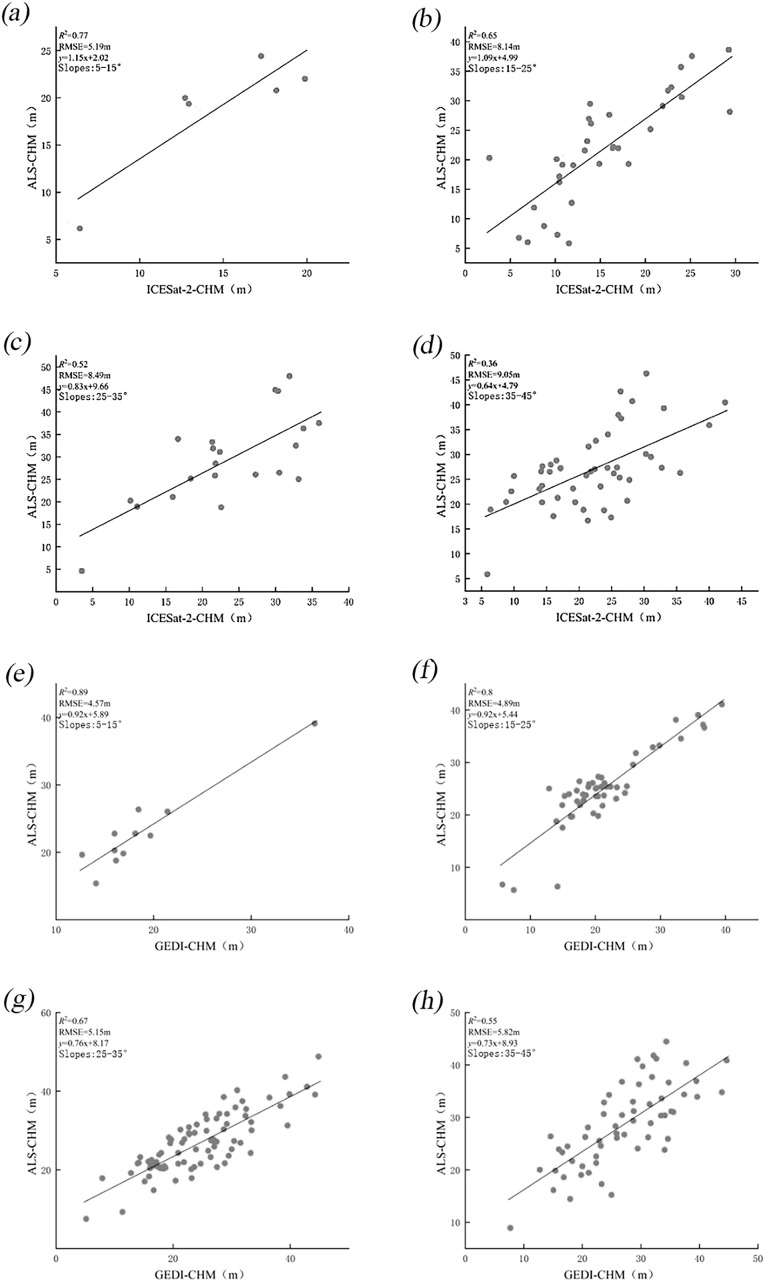
Scatter plots of CHM from ICESat-2 **(a–d)** and GEDI **(e–h)** compared to airborne LiDAR after scale unification, across gentle, moderate, steep, and extremely steep.

Extensive research (Fayad et al., 2021a; Quiros et al., 2021; Mulverhill et al., 2022; Huang et al., 2023b) has demonstrated the significant impact of topographic conditions on the accuracy of forest canopy height measurements obtained from ICESat-2 and GEDI spaceborne lidar. Nonetheless, there is a lack of detailed analysis regarding the performance of these measurements under different slope conditions. Therefore, this section investigates and evaluates the accuracy of canopy height measurements from these two spaceborne lidar systems under varying slope influences, all while using a consistent scale. Scatter plots of the two spaceborne lidar systems under different slope conditions are presented in **Figure 8**, with detailed data available in **Table 4**.

**Table 4:** Results of CHM accuracy verification for different slopes after scale unification.

Gradient Scale	ICESat-2		GEDI	
	R^2^	RMSE/m	R^2^	RMSE/m
Gentle slope (5-15°)	0.77	5.19	0.89	4.57
Moderate slope (15-25°)	0.65	8.14	0.80	4.89
Steep slope (25-35°)	0.52	8.49	0.67	5.14
Extremely steep (35-45°)	0.36	9.05	0.55	5.82

After unifying the measurement scales, the accuracy of ICESat-2 measurements has significantly improved, while the performance of GEDI has slightly declined. This aligns with the terrain height inversion analysis presented in Section 3.1, where it was found that, following scale unification, ICESat-2 results were enhanced, whereas GEDI measurements were reduced. As the slope increases, R² values for both datasets decrease, and RMSE values increase, indicating that slope plays a significant role in influencing the forest canopy height measurements obtained from the two spaceborne LiDAR datasets. On relatively flat terrain, the canopy height measurements from ICESat-2 and GEDI show minimal differences. However, when the slope exceeds 15°, measurement errors for both datasets increase, and accuracy decreases. Notably, when the slope exceeds 35°, the occurrence of abnormal values rises. This may be due to the impact of steep slopes on LiDAR signal reflection and propagation, which complicates ground echo detection and leads to higher measurement errors. Therefore, for high-precision measurements, data from regions with steep slopes should be minimized.

### 3.3 30m resolution CHM mapping in the study area

In summary, the accuracy of DEM and CHM data from the two spaceborne lidar systems, regardless of whether the terrain is flat or steep, or whether scale unification has been applied, indicates that ICESat-2 provides better DEM accuracy than GEDI. Conversely, GEDI exhibits superior CHM inversion accuracy compared to ICESat-2, as CHM accuracy is less influenced by slope and exhibits more stable RMSE values. Consequently, this study selects GEDI L2A data for mapping 30m resolution CHM within the study area.

**Figure 9:**
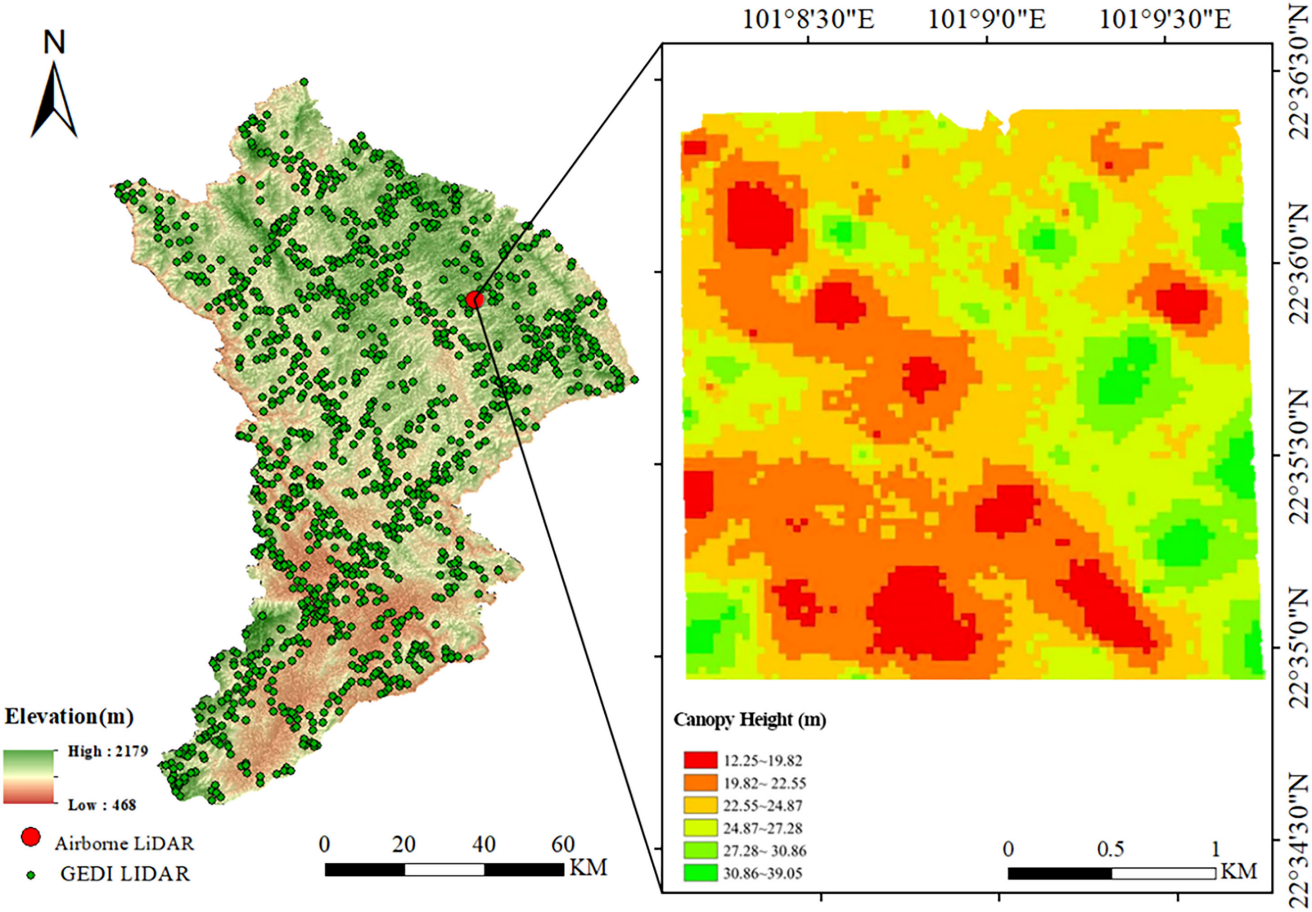
Study area forest canopy height map with 30m resolution.

**Figure 10:**
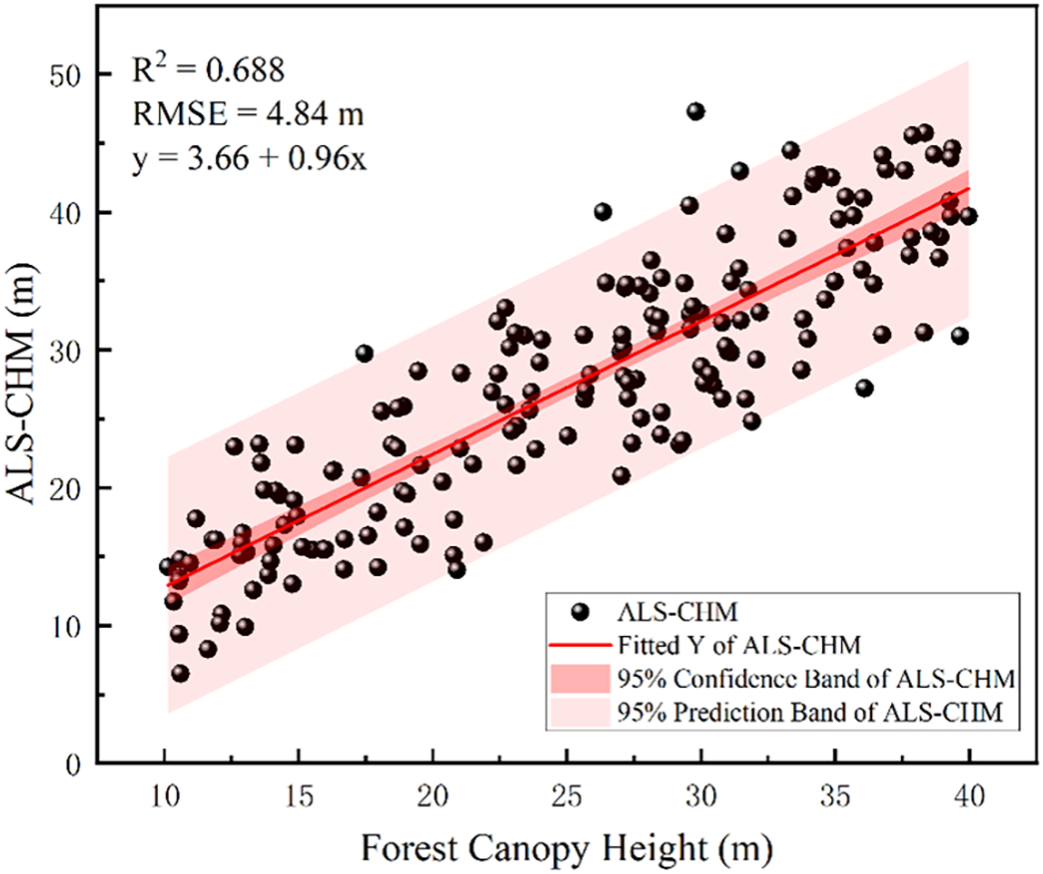
Interpolation spot accuracy verification results.

In the analysis of the variogram for forest canopy height, to meet the normality assumption of the Sequential Gaussian Simulation (SGCS) method, a square root transformation was applied to the RH90 data from 181 GEDI L2A footprints, enabling a better approximation of a normal distribution. GS+9 software was then employed to fit the variogram using Gaussian, spherical, and exponential models. The results (**Table 5**) showed that the exponential model offered the best fit (R²=0.532, RSS=0.057), making it the preferred choice for the subsequent ordinary kriging interpolation. Additionally, 50 simulations were found to be the optimal number for SGCS to ensure stable results. Following this procedure, a 30-meter resolution map of forest canopy height for the study area was generated (**Figure 9**). To assess the accuracy of the interpolated canopy height at GEDI L2A footprint locations, the estimated values were compared with corresponding airborne LiDAR CHM data. The validation results (**Figure 10**) provided an R² of 0.688 and an RMSE of 4.84 meters. These findings are consistent with the GEDI L2A canopy height validation results both before and after scale unification, confirming that the ordinary kriging-based sequential Gaussian simulation interpolation method delivers reliable predictions for forest canopy height.

**Table 5:** Variogram model fitting parameters.

Model	R²	RSS	Nugget	Sill	Variance/%	Range/m
Spherical Model	0.363	0.078	0.001	0.584	0.17	148
Gaussian Model	0.375	0.077	0.056	0.585	9.57	128
Exponential Model	0.532	0.057	0.147	0.60	24.5	369

## 4 Discussion

### 4.1 Analysis of DEM and CHM validation results

The results indicate that, although both GEDI and ICESat-2 exhibit strong terrain inversion capabilities, they show notable differences in terrain height estimation (**Figure 5**). Specifically, ICESat-2 demonstrates a lower RMSE than GEDI, suggesting consistent performance in terrain assessment. When using satellite laser altimeter data to generate terrain height products, it is crucial to note that accuracy depends on terrain complexity, with gentler slopes producing more accurate results compared to steeper slopes, a finding that aligns well with (Liu et al., 2021). Among the RH metrics extracted from GEDI and ICESat-2 data, RH90 has the highest correlation with canopy height obtained from airborne LiDAR. This contrasts with (Zhu et al., 2022), where RH values were reported as 95 and 100. The discrepancies are likely ascribable to the unique combination of vegetation types, forest structural characteristics, and complex terrain existing in the southwestern region of China, which markedly differ from those in the study area in the United States as indicated in (Zhu et al., 2022). These differences may affect LiDAR signals, leading to variations in the correlation between the RH index and canopy height. This indicates that the optimal canopy height percentile for GEDI and ICESat-2 data may vary due to differences in elevation and forest type. The accuracy validation results of this study are relatively low, likely due to several factors. First, the study area is characterized by complex terrain with substantial slope variations. Second, the airborne LiDAR data used are limited in range, and spaceborne LiDAR data within this range are sparse. To increase the data volume, the time frame for acquiring spaceborne LiDAR data would need to be extended. However, this extension would introduce a temporal mismatch with the airborne LiDAR data, potentially affecting the accuracy validation results. Third, in spaceborne LiDAR technology, the energy emitted by the beam directly affects its capacity to penetrate the vegetation canopy. Moreover, atmospheric conditions and noise levels can fluctuate based on the timing of data collection. It is important to note that the energy output of ICESat-2’s high-powered beam and GEDI’s full-strength beam are 4 times and 3.3 times greater, respectively, compared to their weaker and less powerful counterparts (Liu et al., 2021). This suggests that surface height data obtained using strong beams should theoretically offer higher accuracy.

### 4.2 Accuracy analysis of DEM and CHM before and after scale unification

This study represents the first attempt to achieve consistency in scale between ICESat-2 and GEDI data for a comprehensive assessment of terrain and forest canopy heights. This innovative approach is unprecedented in the field of satellite laser altimeter accuracy evaluation and is expected to significantly improve measurement precision. The research results demonstrate that the LiCHy footprints, along with the ATL08 and L2A products prior to and after scale unification, exhibit a significant consistency in relation to the calculated average terrain elevation and canopy height. Meanwhile, the results indicate that the validation accuracy of the DEM and CHM obtained from the ICESat-2 ATL08 data product is R² = 1 and RMSE = 4.75 m, and R² = 0.65 and RMSE = 7.42 m, respectively. After scale unification (30×30m), the DEM and CHM validation accuracy is R²=1 and RMSE = 4.21m, and R² = 0.53 and RMSE = 8.29 m, respectively. These findings suggest that the experimental method for scale unification of ICESat-2 can further enhance the validation accuracy of DEM, reducing RMSE by 0.54 m. This improvement is attributed to the increased resolution of the ICESat-2 ATL08 product, which allows for more frequent sampling of the ground and provides more detailed topographic information. Consequently, this higher sampling frequency enhances the accuracy of terrain detail capture, ultimately leading to reduced errors (Neuenschwander and Pitts, 2019). However, it results in a decrease in CHM accuracy. It is noteworthy that the CHM inversion results of ICESat-2 for each slope are better after the unification of measurement scales than before. This may be because the data are more consistent during processing and analysis after scale unification, reducing errors caused by scale differences and enhancing the inversion accuracy for each slope. However, the overall CHM accuracy verification results are affected by multiple factors in a comprehensive manner and are not simply determined by the situations of each slope interval, especially under complex terrain conditions (Moudrý et al., 2022).

The observed decrease in accuracy may be attributed to the limited number of photons reaching the ground under dense forest conditions with ICESat-2. Consequently, ATL08 products may fail to accurately identify ground photons, or the identified photons may be insufficient to represent the terrain beneath the canopy. This results in lower accuracy in estimating canopy height in dense forests (Neuenschwander and Magruder, 2016). It suggests that, in regions with high vegetation coverage or significant slope variations, scale unification may not yield the expected improvement in accuracy. Instead, the overall accuracy is likely to decrease due to these complex conditions. This discrepancy underscores the impact of scaling effects on characterizing terrain relief heterogeneity, with accurate representation of relief variability being critical for obtaining reliable canopy height measurements.

The validation accuracy of the DEM and CHM derived from the spaceborne LiDAR GEDI L2A data products is R² = 1 with RMSE = 4.94 m and R² = 0.73 with RMSE = 5.15 m, respectively. After scale unification, the validation accuracies for DEM and CHM are R² = 1 with RMSE = 4.96 m and R² = 0.67 with RMSE = 5.32 m, respectively. These results suggest a decrease in accuracy following scale unification, with RMSE values increasing by 0.02 m and 0.17 m, respectively. This decline in accuracy is likely because GEDI’s native 25m footprint captures finer-scale variations. Unifying to a larger 30m × 30m grid averages measurements over a more heterogeneous area, smoothing out these details and reducing accuracy.

### 4.3 Effect of slope on the accuracy of DEM and CHM

Research indicates that the slope of the terrain significantly affects the accuracy of elevation estimates from ICESat-2 and GEDI, both before and after scale unification. This observation aligns with the results from (Zhu et al., 2022), which indicated that slope has the most significant effect on the accuracy of terrain height retrieval. Additionally, study (Urbazaev et al., 2022) highlighted that slope is the primary factor influencing the precision of elevation estimates obtained from ICESat-2 and GEDI. When working with terrain height products derived from spaceborne LiDAR data, it is crucial to recognize that the intricacy of terrain features directly impacts measurement accuracy. Gentle slopes generally yield more accurate results than steep slopes (**Figure 5**). When the slope is within the range of 0 to 35°, ICESat-2 data provides better ground elevation accuracy compared to GEDI data. However, when the slope is 35° or greater, GEDI data exhibits superior ground elevation accuracy compared to ICESat-2. This impact may be due to the photon classification algorithm of ICESat-2 ATL08, which is more susceptible to noise interference in steeper areas. As a result, with increasing slope, the number of retained noise photons increases, leading to a decline in photon classification accuracy.

Slope remains a significant error factor in canopy height estimation (**Figure 7**, **Figure 8**). The error in canopy height increases with the slope. For slopes less than 15°, the RMSE of ICESat-2 and GEDI is both less than 5.2 m before and after scale unification. However, for slopes greater than 35°, the RMSE of ICESat-2 increases to 7.45 and 9.05 m, respectively, while GEDI’s RMSE rises to 5.35 and 5.82 m. This discrepancy may be attributed to the fact that, in areas with steeper slopes, GEDI’s laser signals reflect both vegetation and ground information. This makes it challenging to accurately distinguish ground elevation from the echo waveform, thereby increasing surface errors and reducing the accuracy of CHM. Additionally, the mixing of ground and vegetation echo signals causes waveform confusion, as slope increases, the inversion accuracy of canopy CHM decreases accordingly. Therefore, removing low-quality and high-slope (greater than 35 degrees) waveform and photon data when using GEDI and ATL08 data for forest canopy height inversion can improve accuracy.

The study also found that ICESat-2’s capability to invert forest canopy height is lower than GEDI’s in complex terrains. This may be due to the high forest cover and multi-layered canopy in regions like Jinghong and Pu’er, which are tropical rainforest areas. The ATL08 products of ICESat-2 might struggle to correctly identify ground photons, leading to significant errors in canopy height indices. Hence, when estimating forest biomass using canopy height products from ICESat-2 and GEDI, the GEDI dataset should be preferred (Dorado-Roda et al., 2021; Shendryk, 2022; Wang et al., 2024).

## 5 Conclusions

In this study, the DEM and CHM derived from UAV airborne LiDAR data were used as reference values. The accuracy of both DEM and CHM was evaluated both before and after standardizing the data scales of ICESat-2 and GEDI spaceborne LiDAR in regions with complex terrain. Furthermore, a slope analysis was conducted to investigate how slope influences canopy height inversion. The GEDI L2A data, which demonstrated superior CHM accuracy, were used to generate a 30-m resolution forest canopy height map for the study area using the sequential Gaussian simulation method with Simple Kriging interpolation. The results indicate that:

1. Before and after scale unification, consistently strong correlations (R² = 1.00) were observed between airborne LiDAR DEMs and spaceborne LiDAR DEMs. This strong correlation, even in complex terrain, highlights the fundamental capability of both ICESat-2 and GEDI for terrain representation, consistent with findings in North American mountainous regions (Liu et al., 2021). However, while scale unification reduced the RMSE of ICESat-2 DEMs from 4.75m to 4.21m, GEDI’s RMSE slightly increased from 4.94 m to 4.96 m, confirming that ICESat-2 DEM accuracy remains superior to GEDI in this study. Therefore, for DEM inversion, ICESat-2 data should be prioritized, especially when high accuracy is required in complex terrain.
2. DEM accuracy for both spaceborne LiDAR systems exhibits a negative correlation with slope. For slopes below 15°, the RMSE difference is minimal, and both satellite LiDAR systems offer relatively accurate DEMs, indicating their suitability for broad-scale terrain mapping in gently sloping regions, as evidenced by applications in (Adam et al., 2020; Neuenschwander et al., 2020). However, while ICESat-2 generally offers better ground elevation accuracy than GEDI data for slopes between 0° and 35°, GEDI demonstrates superior accuracy at slopes exceeding 35°. This slope-dependent accuracy crossover underscores the importance of sensor-specific selection based on terrain characteristics, a consideration further emphasized by (Liu et al., 2021).
3. As the canopy height percentile increases, the R² values of GEDI and ICESat-2 data with respect to airborne LiDAR measurements initially rise, then fall, while RMSE values first decrease, then increase. At the RH90 canopy height percentile, GEDI and ICESat-2 data show the highest correlation with airborne LiDAR measurements of forest canopy height, with R² values of 0.73 and 0.53, and RMSE values of 5.15 m and 8.29 m, respectively.
4. GEDI consistently outperforms ICESat-2 for canopy height inversion, regardless of scale unification, and exhibits lower sensitivity to slope, consistent with (Li et al., 2023b). When the slope is less than 15°, the difference in canopy height measurements between ICESat-2 and GEDI is negligible. However, when the slope is 15° or greater, ICESat-2’s CHM measurements exhibit larger errors, with RMSE values ranging from 8 to 10 m. This suggests that for canopy height mapping in complex terrain, GEDI L2A data is generally preferable, especially when slopes exceed 15°. However, users should be aware of the RMSE range of 5-10m for CHM in sloped terrain even with GEDI, indicating limitations for applications requiring very high CHM precision, as also discussed in (Rajab Pourrahmati et al., 2023).
5. A 30-m resolution forest canopy height map for the study area was generated using sequential Gaussian simulation with Simple Kriging interpolation. Validation with airborne LiDAR demonstrated good interpolation results, with an R² value of 0.69 and an RMSE of 4.84 m. These findings underscore the significant potential of next-generation full-waveform LiDAR (GEDI) for producing large-scale, high-resolution forest canopy height maps.

This study lays the foundation for the efficient use of ICESat-2 and GEDI data in estimating terrain and canopy height, while also investigating the effect of slope on the accuracy of data. A positive correlation between slope and error magnitude was observed, indicating that as slope increases, the magnitude of error also increases. In terms of ground elevation retrieval accuracy, ICESat-2 demonstrates relatively high precision, while GEDI excels in vegetation canopy height retrieval. However, due to the geographical constraints of GEDI, which is predominantly concentrated in mid-to-low latitudes, additional data sources are necessary for areas outside the 51.6° north and south latitudes. In this context, this study provides key insights into the significant impact of slope on the accuracy of terrain and canopy height products derived from GEDI and ICESat-2, providing valuable accuracy estimates for users of satellite-based laser altimeters in sloped terrain. Future research should focus on developing slope-adaptive correction algorithms for spaceborne LiDAR DEMs and CHMs, and exploring advanced data fusion techniques to integrate ICESat-2 and GEDI with optical and Synthetic Aperture Radar (SAR) data for improved terrain and canopy height mapping.

## Data Availability

The raw data supporting the conclusions of this article will be made available by the authors, without undue reservation.
