# Supplementary material for: Accuracy assessment of topography and forest canopy height in complex terrain conditions of Southern China using ICESat-2 and GEDI data
Source: Front Plant Sci. 2025 Mar 20;16:1547688. doi: 10.3389/fpls.2025.1547688 (PMC11965696; doi:10.3389/fpls.2025.1547688)
Supplement: Supplementary file 1 [file Table1.docx]

Supplementary Material

**Table S1.** The key mission parameters of ICESat-2/ATLAS and GEDI.

| Parameters | ICESat-2 | GEDI |
| --- | --- | --- |
| Launch date | 2018.09 | 2018.12 |
| Detector type | Photon counting | Full waveform |
| Wavelength | 532 nm (green) | 1064 nm (near IR) |
| Nominal altitude | 500 km | 405 km |
| Ground tracks | 6 | 8 |
| Geographic coverage | 88◦N ~ 88◦S | 51.6◦N ~ 51.6◦ S |
| Footprint size | 17 m | 25 m |
| Along-track | 0.7 m | 60 m |
| Across-track distance | 3.3 km | 0.6 km |
| Laser power  Track number | 120 μJ/30 μJ  6 tracks from 1 laser | 15 mJ/4.5 mJ  8 tracks from 3 lasers |

**TABLE S2.** ATL08 and ATL03 Data Product Extraction Parameter List

| Data Production | Parameter | Brief Description | Parameter Storage Location |
| --- | --- | --- | --- |
| ATL03 | lat_ph | The latitude of each photon point | ATL03/gtx/heights/lat_ph |
| ATL03 | lon_ph | The longitude of each photon point | ATL03/gtx/heights/lon_ph |
| ATL03 | h_ph | The elevation of each photon point | ATL03/gtx/heights/h_ph |
| ATL03 | delta_time | The time at which each photon is recorded | ATL03/gtx/geolocation/ delta_time |
| ATL03 | geoid | Elevation above the geoid (sea level) | ATL03/gtx/geophys_corr/  geoid |
| ATL03 | segment_id | The segment number corresponding to each photon | ATL03/gtx/geolocation/  segment_id |
| ATL03 | segment_length | The length along the track corresponding to each segment | ATL03/gtx/geolocation  /segment_length |
| ATL03 | ph_index_beg | The photon number corresponding to the first photon in each 20 m segment | ATL03/gtx/geolocation/  ph_index_beg |
| ATL03 | dist_ph_along | The photon number corresponding to the first photon in each 20 m segment | ATL03/gtx/heights/  dist_ph_along |
| ATL08 | latitude | The central photon latitude is segmented every 100 m | ATL08/gtx/land_segments/  latitude |
| ATL08 | longitude | The central photon longitude is segmented every 100 m | ATL08/gtx/land_segments  /longitude |
| ATL08 | h_te_mean | Average terrain height per 100 m. | ATL08/gtx/land_segments/  terrain/h_te_mean |
| ATL08 | h_canopy | 98 % relative altitude | ATL08/gtx/land_segments/  canopy/h_canopy |
| ATL08 | ph_segment_id | The index between ATL08 classified photons and each segmented photon point of ATL03 | ATL08/gtx/signal_photons  /ph_segment_id |
| ATL08 | classed_pc_flag | Photon classification identification. 0 denotes noise, 1 denotes ground, 2 denotes canopy photon point, 3 denotes canopy top photon point. | ATL08/gtx/signal_photons/  classed_pc_flag |

**TABLE S3.** GEDI L2A data product extraction parameter list

| Parameter | Brief Description | Parameter Storage Location |
| --- | --- | --- |
| lat_lowestmode | Spot center latitude | /BEAMXXXX//lat_lowestmode |
| lon_lowestmode | Spot center longitude | /BEAMXXXX/lon_lowestmode |
| beam | beam | /BEAMXXXX/beam |
| quality_flag | quality designation | /BEAMXXXX/quality_flag |
| rx_assess_flag | Indicate various error conditions of waveform | /BEAMXXXX/rx_assess/rx_assess_flag |
| degrade_flag | State drop sign | /BEAMXXXX/degrade_flag |
| sensitivity | signal noise ratio | /BEAMXXXX/sensitivity |
| shot_number | Spot identification code | /BEAMXXXX/shot_number |
| elev_lowestmode | surface elevation | /BEAMXXXX/elev_lowestmode |
| elev_highestreturn | canopy height | /BEAMXXXX/elev_highestreturn |
| rh（80,85,90,95,98,100） | Percentile height | /BEAMXXXX/rh |
